# Micro Motion Amplifiers for Compact Out-of-Plane Actuation

**DOI:** 10.3390/mi9070365

**Published:** 2018-07-23

**Authors:** Xin Xie, Majid Bigdeli Karimi, Sanwei Liu, Battushig Myanganbayar, Carol Livermore

**Affiliations:** 1 Mechanical and Industrial Engineering, Northeastern University, Boston, MA 02115, USA; xxie@bwh.harvard.edu (X.X.); majidbk@gmail.com (M.B.K.); lsanwei@gmail.com (S.L.); 2 Massachusetts Institute of Technology, Electrical Engineering and Computer Science, Cambridge, MA 02139, USA; btushig@mit.edu

**Keywords:** microactuators, tactile actuators, piezoelectric actuators, scissor mechanism, motion amplifier, out-of-plane actuator

## Abstract

Small-scale, out-of-plane actuators can enable tactile interfaces; however, achieving sufficient actuator force and displacement can require larger actuators. In this work, 2-mm^2^ out-of-plane microactuators were created, and were demonstrated to output up to 6.3 µm of displacement and 16 mN of blocking force at 170 V. The actuators converted in-plane force and displacement from a piezoelectric extensional actuator into out-of-plane force and displacement using robust, microelectromechanical systems (MEMS)-enabled, half-scissor amplifiers. The microscissors employed two layers of lithographically patterned SU-8 epoxy microstructures, laminated with a thin film of structural polyimide and adhesive to form compact flexural hinges that enabled the actuators’ small area. The self-aligned manufacture minimized assembly error and fabrication complexity. The scissor design dominated the actuators’ performance, and the effects of varying scissor angle, flexure thickness, and adhesive type were characterized to optimize the actuators’ output. Reducing the microscissor angle yielded the highest actuator performance, as it maximized the amplification of the half-scissor’s displacement and minimized scissor deformation under externally applied loads. The actuators’ simultaneously large displacements and blocking forces for their size were quantified by a high displacement-blocking force product per unit area of up to 50 mN·µm/mm^2^. For a linear force–displacement relationship, this corresponds to a work done per unit area of 25 mN·µm/mm^2^.

## 1. Introduction

Actuators apply forces and impose displacements by converting external physical or chemical inputs to mechanical outputs. Larger actuators are typically more powerful than smaller actuators. To the lowest order, force scales with actuator area, and displacement scales with actuator length [1]. Actuators’ reduced outputs at small sizes present a challenge for touch-based technologies like virtual reality, tactile communication, and assistive devices that minimize the impacts of visual impairment. These systems interface with the user’s skin; as such, the force and displacement must be perpendicular to the plane of the actuator. In addition, the force, displacement, and stiffness of the actuator must all be large enough to stimulate the mechanoreceptors of the skin while the area of the actuator is kept as small as possible. The critical performance metrics for touch-based technologies then relate to the stiffness and the out-of-plane force and displacement per unit area, subject to the constraint that the system’s weight, bulk, power draw, spatial resolution, and speed of actuation must also be suitable for practical use.

Vibrating tactile actuators with frequencies of 100–250 Hz were shown to be readily sensed by finger pads for forces of a few mN and for displacements of less than 5 µm [2]. The stiffness of a vibrating tactile actuator that is sensed by the finger pads must also be sufficiently large relative to the finger pads’ stiffness. Finger pads have nonlinear stiffness, with measured stiffness values ranging from a few tenths of a N/mm for small skin deflections to several N/mm for larger skin deflections [3]. If an actuator has lower stiffness than the finger pad does, it will register as being soft, whereas an actuator with greater stiffness will register as being hard. A target stiffness of at least about 1 N/mm is, therefore, appropriate for tactile actuators; this is similar to the stiffness values of the vibrating tactile actuators described by the authors of [2].

Piezoelectric actuators are attractive for these touch-based applications [4,5,6,7] because of their typically low dissipated power and their typically rapid response times, as compared with actuators based on magnetic [8,9,10], electroactive polymers [11,12] or shape-memory alloy [13,14,15,16] phenomena. However, simultaneously achieving large displacements and high forces poses a challenge for piezoelectric actuators. Piezoceramics such as lead zirconate titanate (PZT) offer high forces for their size, but their strains and, therefore, their displacements are very small [17]. Piezoceramics can be engineered to exchange force for displacement, for example, via bending beams [18]. An inherent feature of exchanging force for displacement is either an increased actuator length (for macroscale actuators, as described in References [5,6]) or reduced output force (for thin-film, microfabricated actuators, as described in References [19,20]). Piezoceramics can also be engineered to trade response time for displacement, as described in Reference [7], at the cost of increased system thickness. These tradeoffs limit the resolution and compactness of tactile interfaces based on piezoelectric actuators, such as Braille cells [7], single-line Braille displays [4], and two-dimensional tactile displays for people who are blind or have low vision [5]. 

In Reference [2], a half-scissor mechanism was introduced to ease the tradeoff between force and displacement. The half-scissor converts the small in-plane elongation of a macroscale (bulk) piezoceramic extensional actuator into a much larger out-of-plane displacement that is suitable for vibrational tactile stimulation. Like a conventional scissor mechanism, the half-scissor localizes the deformations at joints (hinges) that connect relatively rigid linkages and supports. This localized deformation distinguishes the half-scissor mechanism from the well-known Moonie and cymbal actuators, in which distributed deformation of an end cap amplifies the deformation of an extensional piezoelectric actuator [21,22]. The challenge of the half-scissor architecture lies in the mechanism’s hinges. The pinned hinges of a conventional scissor mechanism provide ideally localized rotation between the relatively rigid scissor linkages, whereby no spring force opposes the rotation of the ideal pinned hinge. These nearly ideal hinges are readily implemented at the microscale level using silicon surface micromachining [23]; however, the resulting structures are too fragile for touch-based applications. This fragility can be avoided by using flexural hinges, in which a soft or flexible material (often a polymer) approximates ideal joint rotation via more spatially distributed bending along the flexure’s length. In Reference [2], an actuator with a flexure-hinged half-scissor yielded a maximum displacement per unit area of 0.36 µm/mm^2^, a force per unit area of 1.6 mN/mm^2^, and a displacement–force product per unit area of about 15 mN·µm/mm^2^. The actuator’s forces and displacements are suitable for vibrational tactile stimulation. However, the relatively long flexure lengths needed to mimic ideal pinned-hinge behavior in the SU-8 flexures of Reference [2] dictated a large (30 mm^2^) actuator area, limiting the actuator’s application for high-resolution, touch-based communication systems.

**Figure 1:**
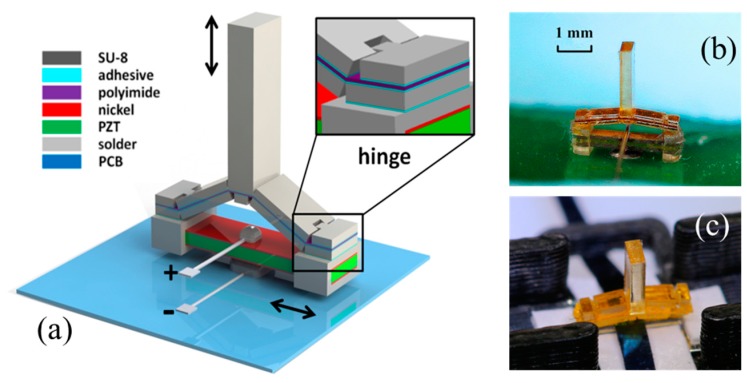
(**a**) Schematic diagram of a motion-amplifying actuator. The system comprises an in-plane extensional lead zirconate titanate (PZT) actuator with a laminated, micro-fabricated half-scissor amplifier to convert small in-plane motions into out-of-plane motions 6–13 times larger; (**b**,**c**) Optical photographs of assembled single tactile elements with electrical connections made (**b**) via soldering, and (**c**) via a scalable packaging approach.

The presented work describes actuators (Figure 1) that are architecturally similar to those of Reference [2], but have an area 15 times smaller (2 mm^2^), while also providing three times as much force per unit area and three times as much force–displacement product per unit area as compared with those in Reference [2]. The actuators presented here expand on Reference [24] by utilizing a self-aligned assembly process and by tailoring the hinges’ mechanics to increase the force output by a factor of three. The small size and enhanced performance of the resulting actuators make them suitable for very high-resolution tactile systems, in which individual elements are spaced between 1 and 2 mm on center [25]. This performance is, in turn, enabled by their compact flexure structure. Specifically, the actuators’ flexures together occupied just 4% of the actuator area while being both flexible enough to offer near-ideal actuation and stiff enough to withstand the forces (on the order of 0.1 N [26]) that are typical of interaction with human skin. Polyimide sheets provided the thin, uniform structures that are required to reliably form these compact flexures. The resulting system’s microscale features, large forces, and overall robustness combine the best of both microsystem and macroscale actuator design.

## 2. Materials and Methods

A schematic diagram of the actuator design is shown in Figure 1, together with photographs of two of the final actuators. The in-plane actuation was provided by a bulk piezoceramic (PZT) beam that expanded and contracted along its length when a voltage was applied across its thickness, as in Reference [2]. These extensional actuators were custom-cut, y-poled PZT beams with dimensions of 3 mm × 0.67 mm × 0.38 mm (Piezo Systems, Inc., Woburn, MA, USA); they were poled uniformly in the vertical direction (y-poled) to couple a vertical electric field to in-plane extension. The capacitance of the PZT beam was 0.098 nF. The force that the beam could apply in expansion and contraction was much larger than the forces required for a vibrating tactile actuator, which were measured at a few mN to <10 mN in Reference [2], depending on frequency. However, its displacement amplitude was much smaller than the approximately 4-µm deflection that was able to be robustly sensed in Reference [2]. The bulk actuator’s output was converted into useful, out-of-plane forces and displacements by coupling it to a microelectromechanical systems (MEMS)-enabled half-scissor mechanism comprising two layers of lithographically patterned SU-8 epoxy microstructures, laminated with a thin film of polyimide and adhesives. The final structure comprised the microscissor mounted on the ends of the extensional actuator.

The thicker, photolithographically defined SU-8 elements formed the rigid linkages. When the linkages were laminated on either side of the polyimide film, flexural hinges formed in the gaps between the SU-8 elements, where the film (Young’s modulus of about 2 GPa) was free to bend. With the microscissor mounted on the extensional actuator, the contraction and expansion of the actuator bent the scissor’s hinges so that the center of the mechanism rose and fell. The amplification factor between the in-plane and out-of-plane displacements was primarily set by the initial angle between the linkages and the PZT beam [2]. Angles of about 9° and 4.5° were used here, corresponding to nominal amplification factors of about six times and 13 times, respectively, although a too-stiff mechanism can reduce the amplification and the final displacement. The offset, interleaved (castellated) hinge structure, as described in Reference [27], minimized hinge deformation apart from the intended bending. The present hinge structure shortened the flexure length as compared with the devices of Reference [2], both in absolute terms and as a fraction of total actuator length, maximizing the space available for longer linkages that increased vertical displacement.

Although the tactile actuators examined here all had the same linkage design, their final materials, dimensions, and assembly and packaging processes were varied from device to device, in an effort to demonstrate the effects of each parameter on actuator performance. The detailed specifications of the as-implemented actuators are summarized in Table 1. The thicknesses of the flexural hinges and the hinges’ starting angles were varied to examine the effects of hinge mechanics on actuator performance. The devices are designed for and assembled using both an actively aligned process and a passively aligned process. The adhesive that bound the linkages to the polyimide flexure layer was varied between a low-modulus silicone adhesive and a stiffer, cyanoacrylate adhesive. Finally, the devices were manufactured using both single-unit and scalable packaging processes. Two nominally identical devices were included to confirm repeatability.

**Figure 2:**
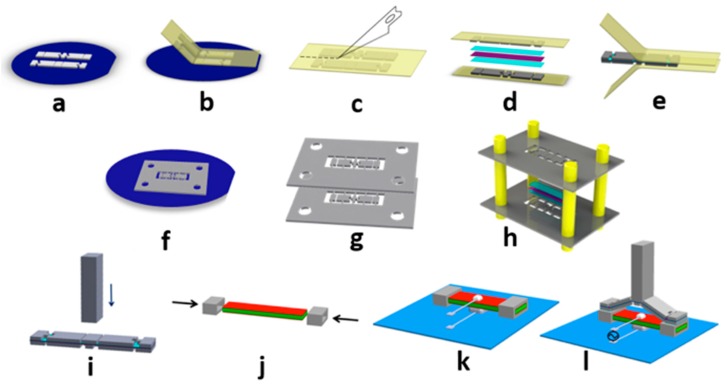
Fabrication process for the motion-amplifying half-scissor actuator. Images (**a**–**e**) show the actively aligned assembly, including (**a**) photolithographically patterning a layer of 200-µm-thick SU-8 2150 on a wafer substrate coated with a 15-nm-layer of Omnicoat™ release layer; (**b**) transferring the SU-8 parts from wafer to polyimide carrier tape by dissolving the Omnicoat™ layer; (**c**) separating the upper and lower sets of SU-8 parts; (**d**) laminating the polyimide film, adhesives, and both sets of SU-8 parts; and (**e**) peeling off the polyimide carrier tape; Images (**f**–**h**) show the passively aligned assembly, including (**f**) photolithographically patterning the linkages in supporting frames; (**g**) releasing the frames from the wafer; and (**h**) aligning the assembly of the scissor elements onto a polyimide tape; Images (**i**–**l**) show the final assembly via solder-based packaging, including (**i**) attaching the tactile interface pin on the central stage of the motion amplifier; (**j**) attaching the SU-8 clamps (fabricated separately) to the PZT extensional actuator; (**k**) soldering the PZT’s upper and lower electrodes to the printed circuit board; and (**l**) adhering the motion amplifier to the PZT actuator to complete the device.

The micro motion amplifier was fabricated using a combination of microfabrication (photolithography) and lamination assembly as illustrated in Figure 2a–e. In the actively aligned process, the relative positions of the as-fabricated linkages were retained by firstly laminating them onto a temporary adhesive “carrier tape”, then releasing them from the fabrication substrate and laminating them onto the polyimide flexure layer under a stereomicroscope. For the actively aligned assembly, both the upper and lower rigid structures were patterned in 200-µm-thick SU-8 2150 epoxy (MicroChem Corp., Westborough, MA, USA) over an Omnicoat™ release layer (MicroChem) using a single-layer photolithography process on a silicon wafer. The carrier tape was made of polyimide with a 38-µm (1.5-mil) layer of silicone adhesive (DC-KHN966 Dupont Kapton^®^ Polyimide with 966 Adhesive, TapeCase Ltd., Elk Grove Village, IL, USA), and it was adhered to the SU-8 structures while they were still on the wafer. Dissolving the Omnicoat^TM^ in Remover PG (MicroChem) then released the SU-8 structures from the wafer while preserving their relative positions. The upper and lower SU-8 structures, still on their respective carrier tapes, were then laminated above and below the thin polyimide film that comprised the hinge layer. The SU-8 structures were retained on the polyimide hinge by two layers of adhesive, thereby forming the microscale half-scissor. Two adhesives were used. The first was a 50-µm (2-mil) thick low-modulus silicone adhesive that was pre-laminated on both sides of the polyimide tape (Kapton^®^ 966). The second was an approximately 5-µm-thick layer of cyanoacrylate adhesive (ASI M60) that was dispensed onto the SU-8 parts to adhere them to a pristine polyimide tape (Kapton^®^ polyimide film, Dupont, Wilmington, DE, USA). The dispensed volume was controlled visually to avoid excess adhesive application. Once the layers were adhered, the carrier tape was mechanically peeled off to release the SU-8/polyimide hinge/SU-8 half-scissor structure. For use as a tactile interface, a separately fabricated SU-8 interface pin was then adhered to the central stage of the motion amplifier (Figure 2i). The resulting half-scissors remained straight until they were assembled onto their PZT actuators (Piezo Systems, Inc.).

An alternative method of fabricating the motion-amplifying half-scissor structure was the passively aligned assembly using alignment frames, as shown in Figure 2f–h. The SU-8 patterning for the upper and lower linkage structures was done in the same way as for the actively aligned approach, except that the in-plane pattern defined linkages that were connected to a surrounding alignment frame by fragile tethers. Each frame included four 560-µm-diameter holes through which 540-µm-diameter dowel pins were inserted to enable passive alignment. After patterning, the SU-8 frames were released from the wafer by dissolving the Omnicoat™. With the assistance of the dowel pins, two sets of scissor parts were self-aligned to each other, and bound on opposite sides of the polyimide hinge film. The completed motion amplifier was then released from the frames by breaking the tethers. The passively aligned assembly process offers a path to the parallel manufacture of larger arrays of motion-amplifying half-scissors.

The microscissors were then mounted on the actuators. The overall lengths of the microscissors were designed to be slightly longer than the length of the PZT actuator, by 120 µm. When the ends of the microscissor were aligned with the ends of the PZT actuator, the hinges formed a starting (unactuated) angle of 9° between the linkage and the PZT. A lower angle of approximately 4.5° was also obtained by extending the microscissor beyond the ends of the PZT actuator. 

The half-scissors were mounted on the actuators using two techniques, each of which was designed to enable a different approach to packaging and electrical connection. In the first approach, the actuators were electrically connected by soldering narrow wires directly to the upper and lower electrodes of the PZT actuator, as shown in Figure 2j–l. Because soldered wires do not create a fully planar connection, clearance was required both above and below the PZT actuator. The necessary clearance was implemented by sliding two separately fabricated, hollow, rectangular SU-8 clamps with a wall thickness of 200 µm onto the ends of the PZT actuators prior to the attachment of the microscissors. With the clamps displaced toward the center of the actuator, cyanoacrylate adhesive (ASI M60) was applied to the ends of the PZT beam. The clamps were then returned to the ends of the actuator, where the adhesive ensured that they remained aligned with the ends of the PZT, and the microscissor was attached. The center of the actuator was soldered to a printed circuit board, creating an electrical connection and a mechanical support. The top of the PZT beam was grounded via a second soldered connection. Figure 1b shows a fully assembled actuator created using this approach.

The second packaging approach, shown in Figure 1c, did not require soldering. Instead, an electrical connection from the top and bottom electrodes to the circuit board was provided via metal strips that also acted to mechanically hold the device in place. The lower actuating electrode of a fully assembled actuator without any rectangular clamps at the ends of the PZT beam was connected to an aluminum strip that ran lengthwise along the actuator and connected to the circuit board. The upper ground electrode of the actuator was connected to a 0.1-mm-thick spring-steel strip that ran perpendicular to the actuator, passing between the PZT beam and the microscissor. When the upper metal strip was also threaded through a housing, as shown in Figure 1c, its downward spring force retained the actuator in position, combining electrical and mechanical packaging in a single structure, and potentially enabling larger arrays in a row/column architecture. One disadvantage of this approach is that the need for a housing to interface with the strips potentially increases the spacing between elements of a tactile array.

The characterization of the actuators’ zero-force displacement and blocking force were carried out as described in References [2,24]. Displacement was measured for each actuator using an optical stereomicroscope. The actuators were mounted horizontally, so that their displacements were visible as a lateral motion in the microscope’s viewing plane. The actuator was driven by a square wave bipolar alternating current (AC) voltage with a frequency of 0.5 Hz that was provided by a function generator (Model 4040A, B&K Precision Corp., Yorba Linda, CA, USA), before being amplified and applied to the device. Displacements were measured for voltage amplitudes between 50 V and 170 V, with 10-V increments. Because the maximum peak voltage used was below the PZT’s maximum allowable voltage, the maximum displacements and forces reported here reflect the limits of the test electronics, rather than the limits of the device. While the tip of the actuator moved under the influence of the applied voltage, the microscope camera captured two or three images of the actuator in its maximum and minimum positions for each voltage. The actuator’s displacement was measured by pixel counting in the captured images. The pixel dimensions were calibrated by measuring the width of the tactile interface pin with a digital caliper, and then, by performing a pixel count on the interface pin in the stereomicroscope images. This yielded a per-pixel dimension of 0.45 µm. Displacement values were recorded for voltages at which the pixel count incremented by a full pixel. This avoided interpolation, and the resulting uncertainty corresponded to approximately 10% of the full-scale displacement for a typical device.

To measure the amplitude of the force that the actuators applied when they vibrated in contact with a rigid surface, the individual actuators were mounted on a second testing stage that was, in turn, mounted on the grips of a mechanical tester (5943 Single Column Tabletop Testing System, Instron, Norwood, MA, US). The load cell of the mechanical tester was brought into contact with the actuator’s interface pin until the load cell registered a compressive preload of 40 mN when zero voltage was applied to the actuator. A square-wave bipolar voltage with a frequency of 10 Hz was applied to the actuator, and its peak amplitude was increased from 50 V to 170 V in increments of 10 V. The resulting forces were measured by the mechanical tester’s load cell with an error bar of 1 mN. Because the load cell prevented the actuator’s displacement, the force that the load cell measured was the blocking force at that voltage.

## 3. Results

### 3.1. Fabricated Devices

**Figure 3:**
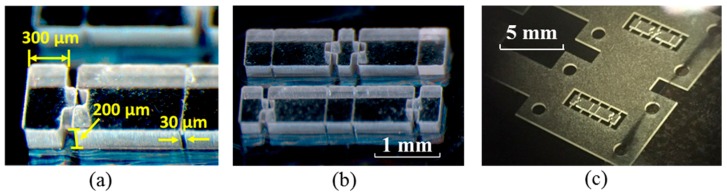
Optical micrographs of (**a**) the 200-µm-thick SU-8 structures (rigid half-scissor linkages for active alignment) with 30-µm microfabricated gaps for hinge bending; (**b**) the upper and lower hinge structures implemented in SU-8 for an actively aligned 3 mm × 0.67 mm microscissor; and (**c**) the 200-µm-thick SU-8 structures tethered on the supporting alignment frames for the passively aligned assembly.

Figure 3 shows the structure of the half-scissors’ rigid linkages. The linkages’ as-fabricated dimensions were 200 µm in thickness, 3.12 mm in length, and 670 µm in width, suitable for the creation of a tactile actuator with an area of 2 mm^2^ after the scissors were bent. In Figure 3a,b, the scissor structures were created for use in the actively aligned assembly process. The benefit of the actively aligned assembly approach was that each microscissor occupied a minimum amount of wafer area; its drawback was the need for visual alignment of the upper and lower components. The structures in Figure 3c were created for a passively aligned assembly process; the SU-8 alignment frames, easily broken tethers, and circular holes in the corners for the passively aligned assembly are visible in the figure. This approach increased the wafer area required for each device; however, it offered a simpler, more rapid device assembly.

Independent of the processes used, the resulting polyimide hinges were 30 µm long, 670 µm wide, and either 25 µm, 50 µm, or 125 µm thick, depending on the thickness of the polyimide film. The lengthwise hinge dimension was set by the microfabricated gaps between the SU-8 components. When silicone adhesive was used, it further increased the thickness of the hinge layer. However, the silicone’s low stiffness reduced its mechanical impact.

### 3.2. Actuator Performance

**Figure 4:**
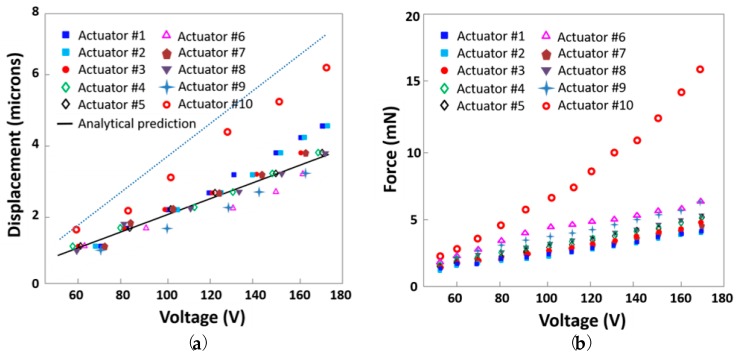
(**a**) Measured peak-to-peak displacement of the actuators as described in Table 1 vs. applied voltage; (**b**) Measured blocking force of the actuators vs. applied voltage.

Figure 4a plots the measured peak-to-peak actuator displacement when voltages of up to 170 V were applied to each actuator; the numbers in the legend correspond to the actuators listed in Table 1. Measurements are represented by markers and can be compared with the displacement predicted analytically using the models of Reference [2] (solid black line for 9° and dashed black line for 4.5°). In all cases, the measured displacement increased approximately linearly with voltage. The lower scissor angle was associated with a higher displacement for a given voltage, as expected given the dependence of the amplification factor on angle [2]. Among the 9° devices, the measured displacement varied depending on the particular hinge thickness and choice of adhesive. The thinnest polyimide hinges yielded the highest displacements. In addition, for a polyimide hinge thickness of 25 µm, the low-modulus silicone adhesive offered greater displacement than the cyanoacrylate adhesive did.

Figure 4b plots the measured blocking force when voltages of up to 170 V were applied to each actuator. The lower scissor angle was associated with much higher values of blocking force for a given applied voltage. Among actuators with the same 9° actuator starting angle, the thinnest polyimide hinge thickness of 25 µm with silicone adhesive produced the lowest blocking forces, which were somewhat increased by the use of thicker hinges or cyanoacrylate adhesive. 

## 4. Discussion

Varying the scissor angle had the greatest effect on actuator performance, with an angle of 4.5° yielding about 30% more displacement than the maximum 9°-device displacement, and greater than three times the blocking force of otherwise comparable 9° actuators. Although the displacement of the 9° actuators generally agreed with the predicted displacement for an ideal pinned-hinge half-scissor, the displacement of the 4.5° actuators lagged behind the predictions. Because the displacement amplification factor of an ideal pinned-hinge scissor is equal to the cotangent of its initial angle [2], halving the scissor angle would be expected to approximately double the measured displacement, in contrast to the observed 30% increase. The predictions assume that the half-scissor’s joints rotate freely like a pinned hinge, with no spring stiffness to be overcome during rotation. In practice, flexure hinges introduce a finite, non-zero stiffness at the joint. In general, this finite stiffness reduces displacement relative to the ideal case. This reduction in displacement for stiffer flexures was observed in the 9° actuators, which displaced less as the flexure thickness increased. It is clear that flexure geometry should affect the stiffness of the joint; however, this does not explain why the displacement at 4.5° was lower than expected, even for devices with otherwise identical geometry.

The reduced displacement at 4.5° can be explained by the bending mechanics of the short, thick flexures. Consider the flexures’ incremental stiffness, which is defined as the ratio of the incremental applied load to the resulting incremental displacement. At small enough bending angles, the flexures’ bending will be purely elastic. As bending angle increases, the flexures’ bending will include increasingly large amounts of plastic deformation. In perfectly plastic deformation, the strain can continue increasing without any further increase in the stress. If the flexures’ bending is approximated as being elastic–perfectly plastic (i.e., with an abrupt transition from perfectly linear elastic behavior to perfectly plastic behavior), the transition to plastic deformation will cause the flexures’ resistance to further bending to decrease. In other words, the flexures will have a lower incremental stiffness at higher bending angles that are well into the regime of plastic deformation than they do at the lower bending angles that leave the flexure predominantly in the elastic deformation regime.

Because the flexures were relatively short (30 µm) compared with their thickness (25, 50, or 125 µm), even bending them to relatively small angles could drive the polyimide into the plastic deformation regime. Achieving a 9° angle would require a radius of curvature of 190 µm for the case of pure bending, corresponding to maximum strains of 6.5%, 13%, and 33% for the 25-, 50-, and 125-µm-thick flexures, respectively. Bending to a 4.5° angle halved the strain as compared with the 9° case, with maximum strains of 3.3%, 6.5%, and 16% for the 25-, 50-, and 125-µm-thick flexures, respectively. The yield transition for polyimide tape occurred at about 3% strain, and the slope of the stress–strain curve remained large up to strains of between 5% and 10% [28]. Bending the flexures to 4.5° retained more of the flexure thickness in the elastic regime than at 9°. For example, if the flexures are approximated as undergoing linear elastic–perfectly plastic deformation in pure bending with a yield transition at about 100 MPa and an elastic modulus of 2.5 GPa, the incremental rotational stiffness of the 50-µm-thick, 4.5° flexure (about 1.1 × 10^−4^ Nm/rad) is predicted to be about seven times greater than the incremental rotational stiffness of the 50-µm-thick, 9° flexure (about 1.6 × 10^−5^ Nm/rad). The less-stiff 9° system more closely matches the predictions for the ideal case, in which the hinge has no stiffness and does not resist bending. 

The increase in incremental flexure stiffness at low angles also explains the three-times-larger blocking force at 4.5° as compared with 9°. For an ideal half-scissor in which the hinges rotate freely, the blocking force would be dominated by the extensional actuator’s piezoelectric stiffness [2]. For the presented actuators, the blocking force was instead limited by deformation of the half-scissor under applied loads. The fact that 9° actuators with thinner hinges exhibited lower blocking forces supports the expectation that flexure bending, rather than piezoelectric stiffness, limits the blocking force of these miniature actuators. As one would expect, the less-stiff 9° system achieved lower blocking forces than the more-stiff 4.5° system. Ultimately, it was the half-scissor’s mechanics, rather than the actuator’s ideal behavior, that dominated the performance of these miniature actuators with flexural hinges. 

Initial bending angle is a key determinant of blocking force; however, device and flexure structure also play a role. The effects of these parameters are visible in the results for the 9° actuators, and they can guide the design choices beyond scissor angle. The thinnest (25-µm) flexures do not permit a high blocking force at any angle. The 50-µm-thick flexures for the 4.5° actuators offered a compromise between achieving adequate elastic stiffness (which requires sufficiently large thickness) and avoiding plastic deformation (which requires sufficiently low thickness). For a polyimide hinge thickness of 25 µm, the low-modulus silicone adhesive (actuators 1 and 2) offered greater displacement than the cyanoacrylate adhesive (actuators 3 and 7), and the cyanoacrylate adhesive effectively increased the actuator’s blocking force. This was as expected, given that the cyanoacrylate adhesive was separately applied and could potentially infiltrate the flexure area. Two otherwise identical actuators that were packaged using the two different mechanical and electrical connection protocols (actuators 1 and 2) showed deviations of at most 6% between their measured displacements and between their measured forces. The consistency between the differently packaged actuators was similar to the, at most, 4% variation between two nominally identical actuators (actuators 4 and 5), indicating that the results are repeatable, and that the packaging technique does not significantly affect the performance of the actuator. 

## 5. Conclusions

In conclusion, we demonstrated the design, fabrication, and performance of a set of compact actuators that had a 15-times-smaller footprint than previous generations of actuators [2], yet still achieved displacements of up to 6.3 µm and blocking forces of up to 16 mN. These forces and displacements are of suitable magnitude for vibrational tactile actuation, as is their corresponding stiffness of about 2.5 N/mm. The maximum peak voltage of 170 V applied here was below the PZT’s maximum allowable voltage, indicating that the maximum measured displacement and force are lower bounds of the actuator’s performance. The presented actuators’ performance was enabled by the use of a laminated hinge architecture that maximized the length available for the scissor linkages in a compact area. The performance of the actuators was shown to depend on the details of the microscissor design, including its angle and the flexure thickness. The highest blocking forces and displacements were obtained from an actuator with a 50-µm-thick polyimide hinge and a 4.5° scissor angle. These maximum values correspond to a displacement per unit of actuator area of 3.2 µm/mm^2^, a force per unit of actuator area of 8 mN/mm^2^, and a force–displacement product per unit area of 50 mN·µm/mm^2^. The force–displacement product is related to the work done by the actuator; for example, for a linear relationship between force and displacement, the work will be half of the force–displacement product. The actuators’ performance compares favorably with the results of Reference [2], which yielded displacement, force, and force–displacement products per unit of actuator area of 0.3 µm/mm^2^, 1.6 mN/mm^2^, and 15 mN·µm/mm^2^, respectively. The presented actuators’ force output per unit area also compares favorably with References [20,29,30], which yielded forces per unit of actuator area of about 1 mN/mm^2^, 1.1 mN/mm^2^, and 0.7 mN/mm^2^, respectively. The presented actuator’s performance may be optimized for various applications, which require various combinations of force and displacement, by varying the layer thicknesses and scissor angles without changing the in-plane design. The consistent performance of the actuators created using passive alignment and via multiple material combinations and packaging options offers simple actuator manufacture that can be adapted to the needs of the application and capabilities of the facility. The passively aligned assembly of the half-scissors also offers a path forward to arrayed fabrication in which multiple half-scissors are manufactured in parallel under the guidance of a common alignment frame; mounting of each half-scissor on its piezoelectric actuator will still require device-by-device assembly. The use of out-of-plane, snap-together mechanical connections, as in Reference [31], that unite the rigid elements around the flexures may further simplify the scissors’ manufacture and enable the creation of larger actuator arrays. Unlike conventional MEMS actuators, which are commonly used for relatively low-force applications like switches and optical devices, these powerful yet compact actuators have significant potential for high-force applications like tactile displays and microrobotics.

## Figures and Tables

**Table 1:** Actuator parameters.

Actuator	Assembly Method	Adhesive Type	Polyimide Thickness	Electrical Connection	Angle
1	actively aligned	silicone	25 µm	metal strips	9°
2	actively aligned	silicone	25 µm	soldered	9°
3	actively aligned	cyanoacrylate	25 µm	soldered	9°
4	actively aligned	cyanoacrylate	50 µm	soldered	9°
5	actively aligned	cyanoacrylate	50 µm	soldered	9°
6	actively aligned	cyanoacrylate	125 µm	soldered	9°
7	passively aligned	cyanoacrylate	25 µm	metal strips	9°
8	passively aligned	cyanoacrylate	50 µm	metal strips	9°
9	passively aligned	cyanoacrylate	125 µm	metal strips	9°
10	passively aligned	cyanoacrylate	50 µm	metal strips	4.5°
